# Federated Transfer Learning Strategy: A Novel Cross-Device Fault Diagnosis Method Based on Repaired Data

**DOI:** 10.3390/s23167302

**Published:** 2023-08-21

**Authors:** Zhenhao Yan, Jiachen Sun, Yixiang Zhang, Lilan Liu, Zenggui Gao, Yuxing Chang

**Affiliations:** Shanghai Key Laboratory of Intelligent Manufacturing and Robotics, School of Mechatronic Engineering and Automation, Shanghai University, Shanghai 200072, China

**Keywords:** federated learning, fault diagnosis, transfer learning, domain adaptation, data privacy

## Abstract

Federated learning has attracted much attention in fault diagnosis since it can effectively protect data privacy. However, efficient fault diagnosis performance relies on the uninterrupted training of model parameters with massive amounts of perfect data. To solve the problems of model training difficulty and parameter negative transfer caused by data corruption, a novel cross-device fault diagnosis method based on repaired data is proposed. Specifically, the local model training link in each source client performs random forest regression fitting on the fault samples with missing fragments, and then the repaired data is used for network training. To avoid inpainting fragments to produce the wrong characteristics of faulty samples, joint domain discrepancy loss is introduced to correct the phenomenon of parameter bias during local model training. Considering the randomness of the overall performance change brought about by the local model update, an adaptive update is proposed for each round of global model download and local model update. Finally, the experimental verification was carried out in various industrial scenarios established by three sets of bearing data sets, and the effectiveness of the proposed method in terms of fault diagnosis performance and data privacy protection was verified by comparison with various currently popular federated transfer learning methods.

## 1. Introduction

With the rapid development of digital intelligent manufacturing, data-driven deep learning methods have made significant progress [1,2]. Various deep learning networks, including computer vision, natural language processing, and autonomous driving, continue to emerge in an endless stream [3,4]. These advancements not only enhance the reliability of equipment utilized in intelligent manufacturing but also improve work safety while reducing maintenance costs [5]. Although deep learning methods alleviate the requirement for operator expertise, the high performance of the network often relies on feature knowledge obtained from a large amount of high-quality training and testing data [6,7].

In practical scenarios, the majority of users in the industrial field possess private condition monitoring data, and there exist analogous mechanical equipment configurations among them [8]. Therefore, amalgamating the condition monitoring knowledge of multiple users to construct a global model for intelligent fault diagnosis can effectively address the issue of insufficient individual user data. However, the device data collected during actual production often contains a significant amount of company-protected device privacy information that is not shared with other users. Thus, centralized data management and centralized fault diagnosis model training for each client are no longer viable [9,10]. In recent years, a federated learning strategy has been proposed to address the issue of collaborative diagnosis among multiple users, effectively mitigating the non-circulation of diagnostic knowledge caused by data privacy concerns [11]. The concept of federated learning was initially introduced by Mcmahan et al. [12], who pointed out that the central server is used to manage the model communication between each client, and the models of each client are averaged. Li et al. [13] proposed a MOON network that leverages model representation similarity to rectify the local training losses of each client, thereby presenting a simplified federated learning framework that effectively addresses the challenge of image heterogeneity adaptation across multiple users. Considering the inherent heterogeneity of local data distribution, Marfoq et al. [14] propose a federated learning strategy for multi-task learning that captures complex relationships among personalized networks through penalty terms.

Despite the preliminary progress made in research on protecting data privacy through federated learning, further advancements are necessary to fully address this issue [15]. Existing federated learning methods often assume that users conform to the same data distribution, meaning that each user collects information on similar mechanical equipment under comparable working conditions. However, in practical scenarios, due to diverse project requirements and distinct operating conditions of industrial equipment, there exist significant discrepancies in data distribution among customers, which pose challenges for the generalization of conventional fault diagnosis methods [16]. Transfer learning breaks the basic assumption that training data and test data must satisfy independent and identical distribution, as it enables the transfer of labeled information from a source domain to diagnose unknown target domain samples [17]. Chen et al. [18] proposed a dual adversarial-guided unsupervised multi-domain adaptation network, which constructed the edge confrontation module (EA-Module) to extract the common features of samples in multiple sets of source domains and validated the method on the transfer task of a rotating machinery dataset. Li et al. [19] proposed a novel joint attention feature transfer network to address the issue of data imbalance in real-world industrial scenarios. Experimental results on the gearbox dataset demonstrate its superior adaptability to sample scarcity.

The existing federated transfer learning model often assumes that each user has stored relatively complete and perfect data sample information when solving multi-user fault diagnosis tasks, which is not common in actual industrial scenarios. Simultaneously, the federated learning strategy does not fully utilize the diagnostic knowledge learned from each source client for other source clients after the global model communication link or completely abandons the accumulated sample diagnostic knowledge of each source client in the local client training link in the current research. 

Referring to the aforementioned issues in current research, this study proposes a federated transfer learning strategy based on data restoration (FTLS-DR). When faced with data damage in the client, this strategy employs linear regression completion on the damaged data as a preliminary step before utilizing it for source client network training. To mitigate any negative transfer effects of broken data on the local model, an offset optimization of the source client network is performed using a joint function composed of maximum mean discrepancy (MMD) and Wasserstein distance (WD). Subsequently, the central server dynamically evaluates the global model based on its performance in task verification for each source client and builds a new round of each source client network by adaptively weighting the global model download link. The main innovations in this paper are as follows:

1. A novel federated learning strategy is proposed to solve the problem that the source client lacks complete samples for network training, which rarely occurs in current federated transfer learning research.
2. The joint function proposed for optimizing source-client networks in federated transfer learning strategies employs Wasserstein distance and multi-kernel MMD to measure domain distances and effectively alleviates the model-negative transfer phenomenon caused by distribution discrepancies through periodic training.
3. To address the challenge of diagnosing targets across different devices and under varying working conditions, an adaptive global model update method is employed by the central server. This approach ensures excellent fault diagnosis performance while safeguarding source client data privacy.

The subsequent sections of this article are structured as follows: Section 2 introduces the related work studied in this paper, while Section 3 presents the network structure and detailed training process of the proposed federated transfer learning strategy. In Section 4, multiple sets of experiments are conducted to discuss the proposed scheme. Finally, Section 5 concludes the entire paper.

## 2. Materials and Methods

### 2.1. Federated Learning

Federated learning was initially proposed as a solution to address the challenge of safeguarding client data privacy in the realm of cross-device fault diagnosis [20]. The framework is designed to facilitate the coordination of network model training among independent parties while ensuring the protection of their respective data privacy [21]. As a distributed machine learning framework, federated learning is divided into three categories: horizontal federated learning (HFL), vertical federated learning (VFL), and federated transfer learning (FTL). Additionally, it mainly includes three sets of training steps: First, the central server initializes the network structure and distributes it along with initial parameter settings to each client. Subsequently, each client utilizes the received network model to perform model training based on local data and uploads the final training result to the central server. Finally, the central server summarizes the client network models of all parties to build a global model with more complete diagnostic knowledge to improve network performance as a whole. 

The training process of the federated learning strategy is distributed to each client, and finally, the aggregation of diagnostic knowledge is realized on the central server, which not only ensures the privacy of all source client users but also promotes knowledge sharing among clients [22,23]. For example, Lee et al. [24] introduced reinforcement learning knowledge into the federated learning strategy and proposed a client selection scheme based on a reward mechanism, which improves the learning efficiency of the network while using fewer agents. Considering that the optimal design of federated learning algorithms in edge computing systems needs to be solved urgently, Li et al. [25] proposed a generalized federated learning strategy that uses the tricks of general inner approximation and complementary geometric programming to iteratively explore the full potential of federated learning. Although significant progress has been made in the aforementioned federated learning methods, there are still numerous challenges that require resolution [26]. This paper further investigates the application of federated learning schemes in few-shot fault diagnosis scenarios.

### 2.2. Transfer Learning

The data-driven deep learning model demonstrates efficient performance in diagnosing faults based on a comprehensive analysis of monitoring data. However, establishing an ideal data set for training deep learning models is challenging in real-world industrial scenarios due to various factors [27]. The main reasons can be summarized in three points: (1) Faults rarely occur under normal operation of mechanical equipment, which makes the collected sample data mostly healthy and free of faulty sample information. (2) The cost of obtaining fault sample information in simulated industrial scenarios within a laboratory setting is relatively high. (3) The fault samples simulated in the laboratory are devoid of environmental information present in real-life scenarios, thereby lacking authenticity.

Transfer learning, as a technique for utilizing diagnostic knowledge from known datasets to address less strongly related fault diagnosis tasks, is highly beneficial for most current domain adaptation methods [28]. For instance, Liu et al. [29] proposed a transfer learning network based on confrontational discriminative domain adaptation to address the fault problem of gas turbines. The approach involves transferring the model trained in the source domain to target domain data, followed by adversarial training that adaptively optimizes model parameters using information from both domains. He et al. [30] proposed a multi-signal fusion confrontation network that integrates vibration and sound signals to diagnose common faults in axial piston pumps. The addition of a multi-signal fusion module enables the re-weighting of each signal, enhancing the accuracy and reliability of fault diagnosis. This study employs the data augmentation method in the transfer learning strategy to enhance the generalization performance of the diagnostic model.

### 2.3. Random Forest

As a fusion strategy of decision tree and bagging methods, the random forest (RF) algorithm constructs a set of low-bias and non-correlation trees (***T*_a_**, a = 1, …, ***R***_tree_) from the predictions given by multiple sets of decision tree models [31,32]. The RF algorithm is often used to solve multi-classification problems and regression problems. When tackling multi-classification problems, the prediction outcomes of all decision trees will be aggregated through voting, and the category with the highest number of votes will be deemed the ultimate diagnostic result. When solving a regression problem, the final prediction will be the mean of all decision tree outputs.

For regression, the mathematical expression of the predicted value given by random forest is as follows [33,34]:

(1) $$\bar{y}(x)=\frac{\sum_{a=1}^{R_{tree}}h(x,\theta_{a})}{R_{tree}}$$

where $h\left(x,\theta_{a}\right)$ stands for the predicted output of the *a*-th decision tree, and ***R***_tree_ represents the number of decision trees in the random forest. This study introduces the random forest algorithm into the federated learning strategy to solve the problem of data corruption in client communication.

## 3. Proposed Federated Transfer Learning Scheme

### 3.1. Network Architecture and Training Initialization

The federated transfer learning strategy proposed in this paper consists of multiple sets of local clients (i.e., multiple sets of source clients and a single target client) and a single central server. To simulate fault diagnosis requirements in realistic scenarios, each source client is assigned a unique diagnostic task that necessitates local data for resolution, while the target client solely possesses target tasks without any training data. Specifically, the local models within each source domain client share identical network configurations as the global model residing on the central server, which is a 3-layer feature extractor and 2-layer classifier network.

Considering that client data privacy needs to be protected, source clients are only allowed to share local model parameters with the central server. In the initialization phase, each source client independently performs model parameter training and diagnostic knowledge learning locally until the maximum set value of training is reached. Subsequently, upon completion of training, the model parameters from each source client are uploaded to the central server for evaluation. The central server then performs weighted aggregation based on the evaluation results of each model to form global model parameters. The federated transfer learning strategy proceeds with initialization until the global model completes its first parameter update.

### 3.2. Source-Client Periodic Training

**Figure 1:**
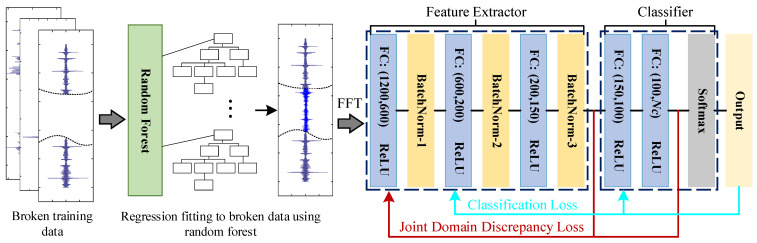
Schematic diagram of source client network architecture and training process.

Considering that the training data contains a large number of diagnostic fault samples, the damaged local training data will be repaired first and then used for the parameter training of the model. The specific local training process and network architecture are shown in Figure 1. 

The random forest algorithm gradually learns the complete part of the training data and performs regression-fitting predictions on the damaged part. In this study, the number of decision trees in the random forest is set to 100, and the number of leaves is 5 groups. At the same time, it is stipulated that the prediction rhythm of predicting 1 point for every 15 points will gradually slip, and the fitting and repair of the damaged data will be completed finally. It is worth noting that “broken data” refers to sample data that loses part of the fragmented information. The repaired time-domain training samples are input to the feature extractor after undergoing a fast Fourier transformation, in which the number of neurons is set to 1000, 800, and 1200. Additionally, the domain discrepancy loss $L_{W}$ function is introduced to solve the problem that there is a significant distribution discrepancy between the training samples and the final fault samples in the target domain. It is worth noting that the Wasserstein Distance (WD) was chosen to evaluate the discrepancy between the datasets [35]. The specific mathematical expression is as follows:

(2) $$L_{W}=\underset{\gamma \in \prod \left(P,Q\right)}{\inf}E_{\left(x,y\right)\sim \gamma }\left[\left\|x-y\right\|\right]$$

where ∏(P,Q) is the set of all joint distributions of the two sets of distributions ***P*** and ***Q***, γ(P,Q) indicates the “mass” that needs to be transported from ***x*** to ***y*** in order to transform the distributions ***P*** into the distribution ***Q***.

The Maximum Mean Discrepancy (MMD) is introduced as the feedback loss $L_{f}$ for local models to optimize the model structure, aiming to mitigate the impact of erroneous diagnostic information extracted from repaired data on network diagnostic performance. Simultaneously, the cross-entropy loss is selected as the sample classification loss of the Softmax classifier, as shown in Formulas (3) and (4):

(3) $$L_{f}\to MMD{\left(P_{S},P_{T}\right)}^{2}=\frac{1}{n_{s}^{2}}\sum_{i=1}^{n_{s}}\sum_{j=1}^{n_{s}}k(x_{i}^{s},x_{j}^{s})-\frac{2}{n_{s}n_{t}}\sum_{i=1}^{n_{s}}\sum_{j=1}^{n_{t}}k(x_{i}^{s},x_{j}^{t})+\frac{1}{n_{t}^{2}}\sum_{i=1}^{n_{t}}\sum_{j=1}^{n_{t}}k(x_{i}^{t},x_{j}^{t})$$

(4) $$L_{c}(o^{s},y^{s})=-\frac{1}{n_{s}}\sum_{i=1}^{n_{s}}\sum_{c=1}^{C}I\left[y_{i}^{s}=c\right]\log\frac{\exp(o_{i,[c]}^{s})}{\sum_{j=1}^{C}\exp(o_{i,[c]}^{s})}$$

where ***k*** is a mapping relationship that maps the original variable to the high-dimensional space, $o^{s}$ and $o^{t}$ represents the features extracted from the source domain and target domain samples, ***I*[·]** represents the probability score of the sample fault type by the softmax classifier.

During the initial phase of source client training, the domain discrepancy loss and the feedback loss are jointly composed of the joint domain discrepancy loss. The local model simultaneously optimizes the joint domain discrepancy loss and the sample classification loss to minimize the domain discrepancy between the source and target domains in the source client task while also utilizing them to rectify model hyperparameters caused by inpainting data offset. The specific mathematical expression is as follows:

(5) $$\underset{\theta_{e},\theta_{c}}{\min}L_{c}(o^{s},y^{s})+\delta 1\cdot L_{W}\left(x_{s},x_{t}\right)+\delta 2\cdot L_{f}\left(o^{s},o^{t}\right)$$

where δ1 and δ4 are empirical coefficients during model training.

The local training in the second stage cancels the optimization of the network parameters by the classification loss. The joint domain discrepancy loss is further optimized to alleviate the negative transfer phenomenon of sample error features to network training caused by random forest regression fitting, and the specific function formula is shown in Equation (6).

(6) $$\underset{\theta_{e},\theta_{c}}{\min}L_{W}\left(x_{s},x_{t}\right)+\delta 3\cdot L_{f}\left(o^{s},o^{t}\right)$$

where δ3 is the empirical coefficient during model training.

The local model is iteratively updated through the continuous joint training of the three sets of objective functions until it reaches the initial preset value. The source client ultimately acquires a set of feature extractors that can effectively capture the relevant information from the fitting data, as well as a set of classifiers capable of distinguishing incomplete feature samples, thereby enabling periodic training for the source client.

### 3.3. Federated Learning Dynamic Interaction

The dynamic interaction process of the federated transfer learning strategy proposed in this paper mainly includes three links: the global model update link, the source client task verification link, and the local model adaptive update link.

**Figure 2:**
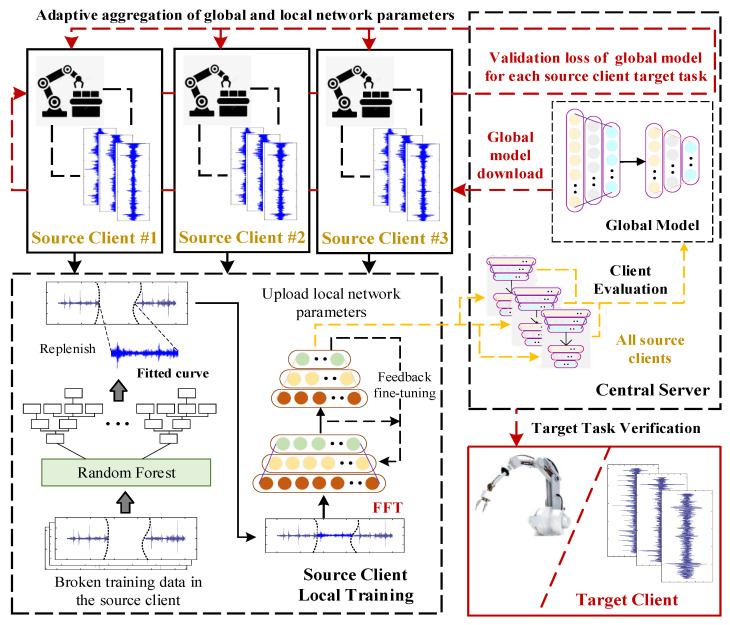
Flowchart of federated transfer dynamic interaction. The red dotted line represents the flow process of the global model, the yellow dotted line indicates the flow process of uploading the local model to the central server, the black dotted line represents the flow process of local model training feedback, and the black solid line represents the local model training process.

The local model parameters from each source client are initially transmitted to the central server, as shown in Figure 2. The central server then assesses the diagnostic knowledge contribution of each source client to the global model and weights and aggregates it to form a new global model network. The functional description is as follows:

(7) $$\lambda_{i,j}=(A_{T}^{i,j}+A_{S}^{i,j})/\sum_{i=1}^{K}\left(A_{T}^{i,j}+A_{S}^{i,j}\right)$$

(8) $$\theta_{E}^{Cen}=\lambda_{1,j}*\theta_{E}^{Client1}+\lambda_{2,j}*\theta_{E}^{Client2}+\cdot \cdot \cdot +\lambda_{K,j}*\theta_{E}^{ClientK}$$

(9) $$\theta_{C}^{Cen}=\left(1/K\right)\cdot \sum_{i=1}^{K}\theta_{C}^{i}$$

where $\lambda_{i,j}$ represents the evaluation coefficient of the *i*-th client in the *j*-th round of federated communication, $A_{T}^{i,j}$ and $A_{S}^{i,j}$ represent the final diagnosis accuracy and training accuracy of the *i*-th client in the *j*-th round of source client training.

Following this, the updated global model is downloaded to each source client for model validation. Specifically, the central server performs reverse verification on all source client tasks one by one and obtains the corresponding sample diagnostic loss to optimize the parameters of the local model, which can effectively improve the ability of the local model to extract the cross-domain universal characteristics of fault samples.

(10) $$\left[z_{1},z_{2},\ldots ,z_{K}\right]=\sum_{i=1}^{K}globel(x_{t}^{i})$$

(11) $$\sum_{k=1}^{K}\left[\theta_{e}^{k},\theta_{c}^{k}\right]\leftarrow L_{c-cen}(label,M(x))=-\sum_{i=1}^{n}label(x_{i})\log\left(M(x_{i})\right)$$

where [z_1_, z_2_, …, z*_K_*] represents the distribution of diagnostic results of the global model of *K*-group source client tasks, $x_{t}^{i}$ is the task verification sample of the *i*-th source client, M(⋅) represents the diagnostic function of the global model, and $L_{c-cen}$ is the diagnostic loss of the global model for the source client task.

In the local model adaptive update link, the parameter information of the global model and the sample diagnostic loss of the source client task are used for a new round of local model parameter updates. Considering the specificity of each source client task, the local model parameters are not completely replaced by the global model. To enhance the generalization performance of local models for cross-domain fault samples while preserving the sample diagnostic knowledge of local tasks, the local model parameters of each source client are adaptively updated, as shown in Formulas (12) and (13):

(12) $$\theta_{e}^{k}\leftarrow H\cdot \left[\left[\theta_{e}^{k},\theta_{e}^{Cen}\right]\cdot \left[A_{T}^{i,j}/\left(A_{T}^{i,j}+A_{cenT}^{i,j}\right),A_{cenT}^{i,j}/\left(A_{T}^{i,j}+A_{cenT}^{i,j}\right)\right]\right]$$

(13) $$\theta_{c}^{k}\leftarrow H\cdot \left[\left[\theta_{c}^{k},\theta_{c}^{Cen}\right]\cdot \left[A_{T}^{i,j}/\left(A_{T}^{i,j}+A_{cenT}^{i,j}\right),A_{cenT}^{i,j}/\left(A_{T}^{i,j}+A_{cenT}^{i,j}\right)\right]\right]$$

where $A_{cenT}^{i,j}$ represents the verification diagnosis accuracy of the *j*-th round of the global model for the *i*-th client task, H[⋅] represents the adaptive update function of the local model parameters.

The three sets of steps of the federation dynamic interaction cycle alternately: the global model gradually masters the fault diagnosis knowledge of all source clients, and the local model of each source client is optimized. Finally, the optimized global model will be delivered to the target client for final verification of the target task.

## 4. Experimental Verification

### 4.1. Dataset Description

In this section, three sets of bearing datasets (including a public dataset and two laboratory simulation datasets) are utilized to validate the efficacy of the proposed method, encompassing three health status categories: normal condition (NC), inner ring fault (IRF), and outer ring fault (ORF). The data set information is shown in Table 1. 

#### 4.1.1. CWRU

The CWRU Bearing Dataset from Case Western Reserve University comprises sample data obtained by the Electromechanical Signal Analyzer at four distinct rotational speeds. The damage diameters of the outer and inner ring faults are categorized as 0.1778 mm, 0.3556 mm, and 0.5334 mm, respectively. In the experiment, the vibration acceleration signal collected by the sensor located at the 6 o’clock position of the motor drive end is selected for research and discussion. Simultaneously, two groups of sampling frequencies are set to 12 kHz and 48 kHz, respectively.

#### 4.1.2. MDS

The Motor Drive Simulation (MDS) Experiment Dataset is collected by the LMS vibration data acquisition instrument at a sampling frequency of 12.8 kHz and a three-way acceleration sensor, specifically the PCB353B33 model. The damage sizes of the outer and inner rings of the bearing are specifically artificial EDM cracks, each with a width and depth of 0.5 mm. Additionally, sample information was collected on the health status of rolling bearings at three different speeds: 1000 rpm, 1300 rpm, and 1500 rpm. The fault samples collected in the time domain are subjected to Fast Fourier Transform (FFT) processing to obtain frequency domain signal samples for training.

#### 4.1.3. GPTFS

**Figure 3:**
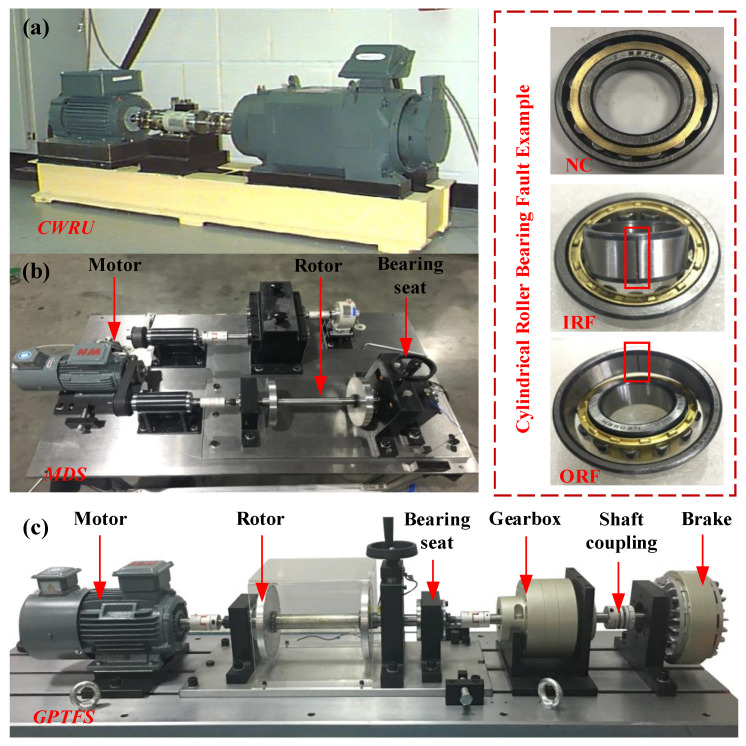
The display diagram of each bearing fault simulation test bench.

The Gear Power Transmission Fault Simulation (GPTFS) Experimental Dataset uses a specially processed cylindrical roller bearing (NU205EM) for experiments and artificially increases crack faults in the outer ring and inner ring of the bearing (i.e., EDM, the crack size is 0.2 mm, 0.4 mm, and 0.6 mm). During the data collection process, the PCB315A acceleration sensor was mounted onto the bearing base and set to a signal collection frequency of 12.8 kHz, and the test bench is shown in Figure 3. Specifically, for the bearing experiment, the data samples were collected from a control motor operating at constant speeds of 1000 rpm, 1500 rpm, and 2000 rpm while also subjecting it to 0 N and 20 N motor loads as per experimental requirements.

### 4.2. Different Comparison Schemes

In order to demonstrate the superiority of the proposed federated transfer learning scheme in addressing the few-shot learning problem, multiple sets of comparative experiments with identical experimental configurations were conducted to validate its effectiveness.

**Baseline**: The **baseline** method [36], which does not incorporate any federated transfer learning knowledge, is commonly employed as a reference group in experiments to assess the reliability and efficacy of proposed schemes. Each source client model performs direct diagnosis on the target task after local task training, and the final diagnosis result for the target task is obtained by aggregating and averaging the results from all source clients.

**FedAvg**: The Federated Averaging (FedAvg) method [37] aims to centrally average the locally trained models and aggregate them into a global model, which is then distributed to each client device through training. This approach achieves the objective of training a shared model with scattered data by employing two stages: local model training and global model aggregation, which **ensure** diagnostic knowledge sharing while preserving data privacy.

**FTLS-DPP**: The Federated Transfer Learning Scheme based on Data Privacy Protection (FTLS-DPP) method is a collaborative strategy designed to address the issue of industrial data islands, with its training process being executed independently on each local client. Specifically, the local model employs differential training to enhance the diagnostic accuracy and generalization of the network, while the global model assesses the task contribution of each local model for weighted aggregation. These two sets of training cycles **alternate** to accomplish the target customer terminal task.

### 4.3. Cross-Machine Federated Transfer Learning Tasks and Parameters Setting

The training process of each source client model is conducted independently in the experiment, thereby ensuring the privacy of individual client data. The complete training of the federated transfer policy does not involve any information regarding the target client tasks, and the detailed experimental task settings are presented in Table 2. Specifically, *K* sets of source clients and a group of target clients were established using bearing samples collected by three sets of test platforms under various working conditions during the experimental verification stage. Each set of clients contains unique fault diagnosis tasks that are consistent with the final target, and there are notable differences in these tasks.

Four groups of samples with missing information and client tasks were established in this study to simulate diagnostic tasks under various working conditions. In the first scenario, each source client sample set contains ideal sample data, and there are discernible discrepancies in the diagnostic tasks of each source client. In the second scenario, not only does the training data contain defects in each client diagnosis task, but it also exhibits a 12.5% rate of sample damage. Furthermore, the federation strategy focuses on more intricate cross-device and cross-type fault sample diagnoses in this scenario. In the third and fourth scenarios, both cross-device and cross-model diagnostic tasks were present in the target client, while load information was also integrated into the data of each source client. By setting up four groups of federated diagnostic tasks, the proposed diagnostic strategy is fully applied. To clarify the operation of the proposed federated transfer learning strategy, the relevant parameter information is established based on the requirements of the target task and presented in Table 3.

### 4.4. Diagnosis Result and Discussion

**Figure 4:**
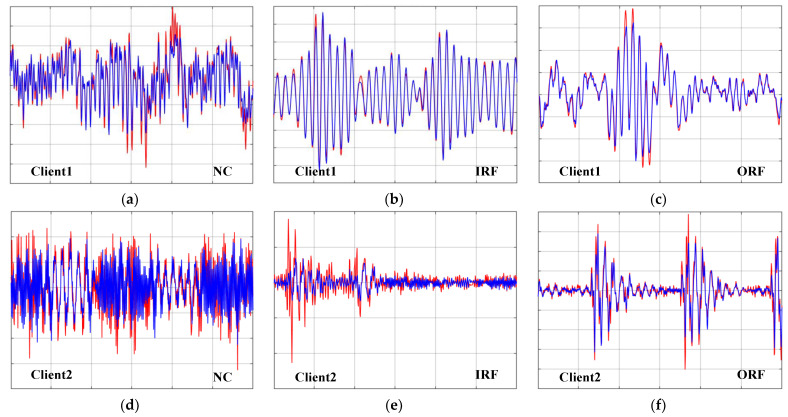

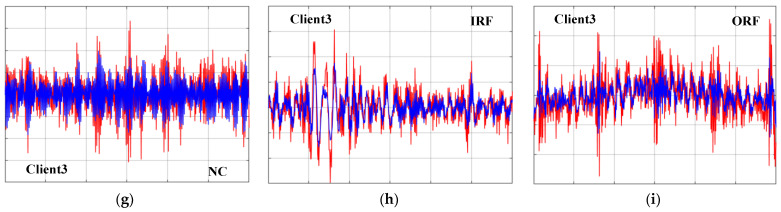
The comparison display of forecast data and real data in case 2: The blue curve represents the predicted data. The red curve represents the real data.

The random forest algorithm is used to perform regression fitting on the damaged training data in the source client, and the fitted data is directly applied to the training process of the network. Figure 4 shows the comparison of the fitting curves of the training samples for each health type of the three groups of source clients in Case 2. It can be clearly seen from the figure that the predicted data for client 1 was constructed by using the CWRU data set, and the real data met a relatively ideal fit, which shows that there are obvious periodic fault characteristics in the data set. As more uncertain environmental interference is mixed into the data set, the peaks of the fitting curves predicted in Client 2 and Client 3 begin to stagger from the real data, but the trend of the fitting curves is always consistent with the real data. 

**Figure 5:**
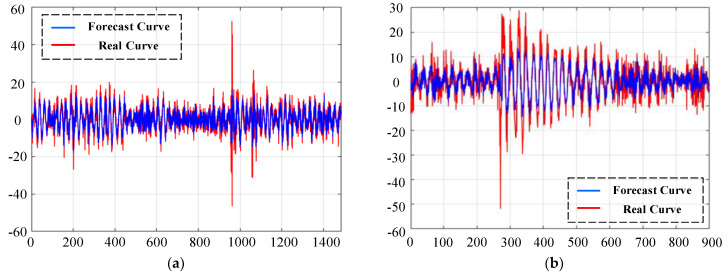
The comparison display of forecast data and real data in case 3 (GPTFS-ORF3-2000 rpm): Figure (**a**) is the forecast curve during model training. Figure (**b**) is the forecast curve during model testing.

Figure 5 shows the comparison between the fitting curve of the outer ring fault sample of the GPTFS data set predicted in the selected case 3 and the real data. Although the trend of the predicted data is basically consistent with the real data, there are still some discrepancies in the magnitude of kurtosis. Given the complexity of the samples in the dataset, the existing prediction bias is allowed during the training of the local model. In the experimental section, a group of damaged fault samples in each case is selected to describe the results of random forest regression fitting. The detailed data restoration indicators are shown in Table 4.

**Figure 6:**
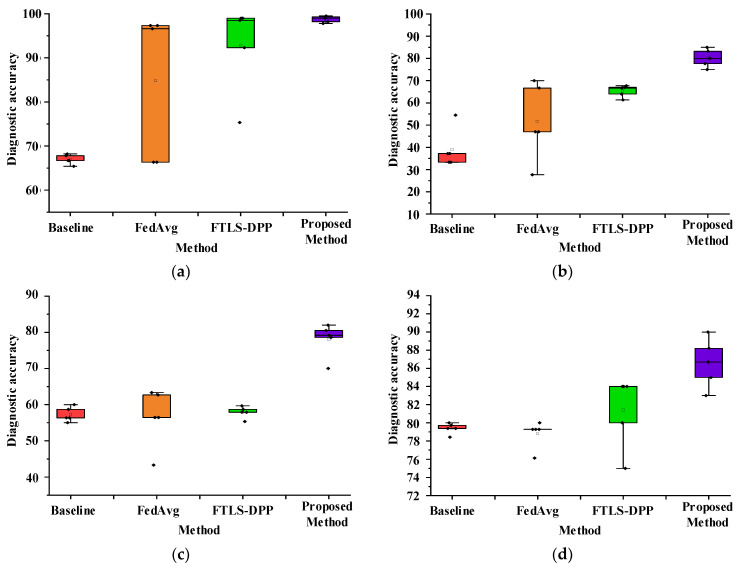
The diagnostic accuracy fluctuation display of each comparison method for the target task. Figure (**a**) is the diagnosis details of the target task in case 1. Figure (**b**) is the diagnosis details of the target task in case 2. Figure (**c**) is the diagnosis details of the target task in case 3. Figure (**d**) is the diagnosis details of the target task in case 4.

In the experimental phase, each diagnostic method was tested five times in each scenario to ensure experiment reliability. The diagnostic accuracy rate and corresponding standard deviation of these comparative experiments are presented in Table 5 and Figure 6.

The FTLS-DR method proposed in this study outperforms other comparison methods in terms of diagnostic accuracy and fluctuation range across all four cases. Specifically, in the case of complete data training in case 1, the diagnostic accuracy of the proposed method in each client task and target client task is higher than 98%, with a standard deviation of 1.04%. Comparing the FedAvg method with the FTLS-DPP method, the diagnostic accuracy is only 84.78% and 92.83%, and the standard deviation is greater than 7.21%. The proposed method still demonstrates superior model generalization performance and diagnostic accuracy, even in the presence of corrupted training data. Specifically, the diagnostic accuracy for the unknown target client diagnosis task remains above 78.06% when 25% of the training data is damaged. It can be inferred that the FTLS-DR method proposed in this paper has better universal feature extraction capabilities for fault samples, rendering it more suitable for diagnostic tasks in complex scenarios.

**Figure 7:**
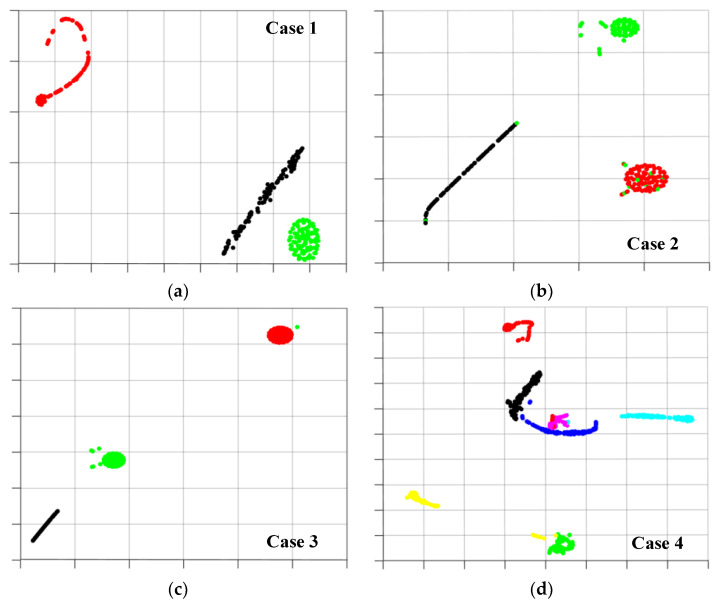
Feature visualization of the target task during testing. The black particles denotes normal condition. The blue particles denotes inner fault 1. The purple particles denotes inner fault 2. The red particles denotes inner fault 3. The yellow particles denotes outer fault 1. The blue-green particles denotes outer fault 2. The green particles denotes outer fault 3.

In order to demonstrate the distribution of features extracted from data samples and validate the advantages of feature extraction using the proposed FTLS-DR method, the high-dimensional features of the target client sample extracted in the final verification link are visualized and displayed through dimension reduction [38], as shown in Figure 7. In cases 1 to 3, each group of clients encompasses three distinct bearing health states, while in case 4, seven bearing health states are set for the problem of misclassification of fuzzy fault samples. The proposed federated transfer strategy still shows satisfactory diagnostic results in the face of fault sample diagnosis under unknown working conditions. From the extracted sample features, it can be seen that the sample data features in each healthy state are accurately extracted and perfectly classified. Except for a small number of fault samples in the outer circle that were misclassified in case 2, there was a staggered phenomenon of individual fault sample cluster boundaries in case 4. This further demonstrates that the FTLS-DR method can still perform satisfactorily in the face of complex transfer tasks across devices and bearing models.

## 5. Conclusions

Aiming at the problem of data privacy protection in actual industrial scenarios, this paper proposes a new cross-device fault diagnosis method based on repaired data. The proposed federated transfer learning strategy is different from the traditional fault diagnosis method. The target client sample does not participate in the network training and parameter updates from the initial training stage to the final target task verification process. Multiple sets of diagnostic tasks are established on three sets of bearing datasets to simulate engineering requirements in real-world scenarios. The results show that the proposed federated transfer learning strategy effectively solves the problem of difficult diagnosis of fault samples caused by the lack of complete local training samples. The proposed FTLS-DR method not only effectively guarantees the privacy of client data but also achieves the best diagnostic results among other comparison methods. In addition, the key indicators and fitting accuracy of the restoration data were measured from multiple perspectives, and the comprehensive evaluation proves that the method has a good prospect for practical engineering diagnosis.

## Figures and Tables

**Table 1:** Information of the three bearing experimental datasets.

Dataset	Object Types	Working Conditions		Health Types	No. of Samples
		Code	Load/Speed		
*CWRU*	SKF6205	A	0 hp/1797 rpm	1 Normal 3 Inner Race 3 Outer Race	100 3 × 100 3 × 100
		B	1 hp/1772 rpm		
		C	2 hp/1750 rpm		
		D	3 hp/1730 rpm		
MDS	NU205EM	E	1000 rpm	1 Normal	100
		F	1300 rpm	1 Inner Race	100
		G	1500 rpm	1 Outer Race	100
GPTFS	NU205EM	H	1000 rpm	1 Normal 3 Inner Race 3 Outer Race	100 3 × 100 3 × 100
		I	1500 rpm		
		J	20 N/1500 rpm		
		K	2000 rpm		
		L	20 N/2000 rpm		

**Table 2:** Detailed information on diagnostic tasks.

	Task	Client #1	Client #2	Client #3	Target Client
Case 1 (3 types)	Source	C (CWRU-1750)	F	K	C, I (CWRU-1750 mixed with GPTFS-1500)
		Ideal Data	Ideal Data	Ideal Data	
	Client task	B (CWRU-1772)	G	I	
Case 2 (3 types)	Source	C	F (MDS-1300)	K	C, I
		Repair Data (25%)	Repair Data (25%)	Repair Data (25%)	
	Client task	B	G (MDS-1500)	I	
Case 3 (3 types)	Source	C	F	K (GPTFS-2000)	C, I
		Repair Data (37.5%)	Repair Data (37.5%)	Repair Data (37.5%)	
	Client task	B	G	I (GPTFS-1500)	
Case 4 (7 types)	Source	C	K	J (GPTFS-20/1500)	D, J (CWRU-1730 mixed with GPTFS-20/1500)
		Repair Data (25%)	Repair Data (25%)	Repair Data (25%)	
	Client task	D (CWRU-1730)	I	L (GPTFS-20/2000)	

**Table 3:** Parameter settings for the proposed scheme.

Parameter	Value	Parameter	Value
Source_input	1200	The number of decision trees $R_{tree}$	100
Target_input	1200	The number of leaves $R_{leaf}$	5
Classification_input	150	$R_{n\_train}$	15
Sample_size	100	$R_{n\_prediction}$	1
Label_1	7	The number of source clients *K*	3
Label_2	3	Experience coefficient δ1	0.5
Learning-rate	0.0005	Experience coefficient δ2	0.5
Sample_size $N_{data}$	100	Experience coefficient δ3	4
Federation dynamic interaction cycle $N_{r}$	20	Local training cycle $n_{k}$	100

**Table 4:** Repair evaluation index of the corrupted training data.

Client Task			*MAE*	*MAPE*	*MBE*	*RMSE*	*R* ^2^
Case 2 (25%)	Client 1	CWRU-NC	0.01684	0.03367	0.00084	0.02243	0.87892
		CWRU-IRF3	0.08165	0.00211	0.00112	0.11341	0.98029
		CWRU-ORF3	0.0298	0.01867	0.00195	0.0432	0.95764
	Client 2	MDS-NC	0.21635	0.05039	0.02098	0.27182	0.73556
		MDS-IRF3	0.49061	0.10457	−0.04878	0.37851	0.65363
		MDS-ORF3	1.0169	0.0381	−0.04772	1.7058	0.84441
	Client 3	GPTFS-NC	2.2796	0.068	−0.11896	2.867	0.66156
		GPTFS-IRF3	2.7295	0.08345	−0.01088	3.8418	0.68568
		GPTFS-ORF3	2.0915	0.10786	0.20306	2.7645	0.63849
Case 3 (37.5%)	Client 1	CWRU-NC	0.01631	0.02180	0.00057	0.02165	0.8835
		CWRU-IRF3	0.08739	0.00142	−0.00014	0.11952	0.97492
		CWRU-ORF3	0.02895	0.01	0.00262	0.03942	0.9602
	Client 2	MDS-NC	0.30154	0.05548	−0.02627	0.42285	0.64347
		MDS-IRF3	0.32726	0.06213	−0.00511	0.41679	0.60667
		MDS-ORF3	1.1004	0.02657	−0.02394	1.7745	0.80962
	Client 3	GPTFS-NC	2.2504	0.048	0.06712	2.8307	0.65488
		GPTFS-IRF3	2.7417	0.0568	−0.031	3.7691	0.68315
		GPTFS-ORF3	1.9348	0.0709	−0.0393	2.5791	0.6464
Case 4 (25%)	Client 1	CWRU-IRF1	0.05333	0.02967	−0.00562	0.08707	0.90167
		CWRU-IRF2	0.01672	0.00638	0.00046	0.02179	0.96908
		CWRU-ORF1	0.10435	0.00504	−0.00899	0.15351	0.97724
		CWRU-ORF2	0.02529	0.02034	0.0025	0.03482	0.95266
	Client 2	GPTFS-IRF1	2.9788	0.0828	0.13673	4.0143	0.69904
		GPTFS-IRF2	2.9474	0.05992	−0.07722	3.6758	0.71246
		GPTFS-ORF1	3.0049	0.06826	−0.1066	3.9895	0.68667
		GPTFS-ORF2	2.0797	0.0932	0.09133	2.7262	0.67296
	Client 3	GPTFS-NC/20N	1.1284	0.0719	0.00471	1.444	0.72461
		GPTFS-IRF1/20N	3.4072	0.098	−0.05176	5.6176	0.60803
		GPTFS-IRF2/20N	1.9464	0.06854	0.06075	2.5784	0.68535
		GPTFS-IRF3/20N	1.825	0.06872	−0.00536	2.2947	0.70688
		GPTFS-ORF1/20N	2.2651	0.07551	−0.0794	2.7943	0.68356
		GPTFS-ORF2/20N	3.2422	0.04868	0.16	4.4067	0.77534
		GPTFS-ORF3/20N	3.1238	0.06493	0.12395	4.1924	0.74757

**Table 5:** Accuracy and standard deviation (%) of the diagnostic results.

Client Task		Baseline	FedAvg	FTLS-DPP	Proposed Method
Case 1	Client 1	88.3 (7.34)	94.36 (5.4)	100 (0)	100 (0)
	Client 2	66.7 (11.67)	74.12 (9.58)	96.9 (3.46)	98.67 (0.2)
	Client 3	54.3 (14.03)	63.66 (3.56)	80.01 (7.63)	100 (0)
	Target	66.96 (1.66)	84.78 (14.76)	92.83 (7.21)	98.76 (1.04)
Case 2	Client 1	40.27 (5.69)	62 (0.22)	75 (6.89)	81.65 (2.34)
	Client 2	38.09 (2.32)	41.78 (2.81)	39.22 (0.74)	78.36 (1.2)
	Client 3	48.45 (5.18)	62.89 (1.26)	65.89 (5.04)	78.44 (2.17)
	Target	39.11 (6.15)	51.67 (13.33)	65.34 (2.14)	80.17 (3.14)
Case 3	Client 1	40.22 (1.59)	43.56 (2.96)	44.89 (5.41)	73.34 (2.48)
	Client 2	61.44 (2.37)	62.67 (3.56)	60.89 (0.95)	67.76 (2.71)
	Client 3	36.67 (4.89)	42.44 (13.92)	29.45 (11.18)	68.35 (3.14)
	Target	57.27 (3.42)	56.44 (5.25)	57.89 (1.03)	78.06 (3.22)
Case 4	Client 1	57.62 (8.44)	58.42 (6.57)	76.28 (11.01)	99.71 (0.17)
	Client 2	67.23 (14.79)	43.57 (21.33)	81 (6.84)	99.88 (0.1)
	Client 3	59.09 (2.89)	71.47 (9.17)	87.14 (4.81)	99.86 (0.15)
	Target	79.38 (0.38)	78.8 (1.06)	81.4 (3.12)	86.58 (2.06)

## Data Availability

The data used to support the findings of this study are available from the corresponding author upon request.
